# What MEMS Research and Development Can Learn from a Production Environment

**DOI:** 10.3390/s23125549

**Published:** 2023-06-13

**Authors:** Malte Florian Niekiel, Jana Marie Meyer, Hanna Lewitz, Anne Kittmann, Marc Alexander Nowak, Fabian Lofink, Dirk Meyners, Jens-Hendrik Zollondz

**Affiliations:** 1 Fraunhofer Institute for Silicon Technology ISIT, Fraunhoferstr. 1, 25524 Itzehoe, Germany; florian.niekiel@dlr.de (M.F.N.);; 2 Institute for Material Science, Kiel University, Kaiserstr. 2, 24143 Kiel, Germany

**Keywords:** process integration, semiconductor manufacturing, microelectromechanical system, magnetoelectric sensor, biomagnetic sensing, process flow, device design, failure mode and effects analysis

## Abstract

The intricate interdependency of device design and fabrication process complicates the development of microelectromechanical systems (MEMS). Commercial pressure has motivated industry to implement various tools and methods to overcome challenges and facilitate volume production. By now, these are only hesitantly being picked up and implemented in academic research. In this perspective, the applicability of these methods to research-focused MEMS development is investigated. It is found that even in the dynamics of a research endeavor, it is beneficial to adapt and apply tools and methods deduced from volume production. The key step is to change the perspective from fabricating devices to developing, maintaining and advancing the fabrication process. Tools and methods are introduced and discussed, using the development of magnetoelectric MEMS sensors within a collaborative research project as an illustrative example. This perspective provides both guidance to newcomers as well as inspiration to the well-versed experts.

## 1. Introduction

Microelectromechanical systems (MEMS)—microscale systems combining mechanical, electrical and other domains—have established their role as a key technology in today’s world [1,2,3,4]. Taking advantage of various effects [5,6], examples of successful MEMS devices include accelerometers and gyroscopes [7,8], pressure sensors [9,10], switches [11,12], laser beam deflection systems [13,14,15,16], flow sensors [17], micropumps [18], microphones and speakers [19,20,21], drug delivery and dosing systems [22,23,24], gas sensors [25] and energy harvesters [26,27,28,29]. With commercial products being mass produced, the MEMS industry has reached maturity and is growing at a fast pace [1,4,30,31]. Nevertheless, groundbreaking research is continuously performed at universities and institutes, yielding an uninterrupted stream of new possibilities and applications [2,32,33,34].

MEMS can be seen as an evolution of microelectronics [1,35], as the availability of a fabrication environment for microelectronics paved the way for the development of the first MEMS devices. While this close relationship is beneficial for the availability of tools and processes, the design of MEMS devices stays challenging. As discussed by Brück et al. [36] and others [35,37,38], the fundamental difference with MEMS is the interdependency between device design and fabrication process due to the geometrical requirements for proper device functionality. While efforts are made to group closely related devices into technology platforms [39,40,41], a fine-tuning of the process flow for each device to meet application requirements is usually unavoidable. The statement “one device, one process” coined by Eloy et al. [30] as *MEMS law* continues to complicate MEMS development. This leads to the management of technology being the key methodology in MEMS design compared to the management of complexity in microelectronics, as elaborated by Brück et al. [36]. Prince et al. [42] conclude that the high cost of design for new devices leads to the industry being dominated by high-volume products. To lower the barriers for MEMS development, Esashi [34] suggests expanding collaboration between industry and academia in prototyping facilities.

With the intricate entanglement of device design and fabrication process, design for manufacturability is the key discipline during MEMS development to reduce time and effort aiming for a first-time-right fabrication. Several groups have worked on this topic reporting different approaches to overcome or mitigate the challenges. Fedder [43] suggests a structured MEMS design method implementing hierarchical partitioning following CMOS design best practices. Watty et al. [44] focus their analysis on the demanding requirements on MEMS designers and provide useful approaches to improve knowledge management. The need for best practices in MEMS design is highlighted by Vudathu et al. [45]. To make a start, they share their approach to apply yield analysis already during the design phase to optimize the robustness of designs with respect to unavoidable process tolerances. Brück et al. [36,38] devise a combined behavioral and physical approach. They introduce the discipline of process management and developed a sophisticated software tool to handle the necessary information. In their assessment on current practices, Sagoo et al. [46] make visible the high number of iterations made in the MEMS development process. They are related to the commonly used expertise-based design approach and the technological limitations of available processes. Prince et al. [42] propose an optimum design method for MEMS development using multiple attribute decision making. Applying parameter sensitivity analysis, the effects on the final device can be evaluated at every stage of the development phase.

Design is not the only task on the road to successful MEMS product development. Ortloff et al. [47] integrate the approach developed by Brück et al. in an overall project management scheme, highlighting the role of the customer (the entity selling the product to the end-customer) contributing important information about constraints and dependencies to be taken into account during product development. Bieniek et al. [47,48] apply this approach exemplarily on the development of an inertial sensor. Wolter et al. [37] provide insight into the design and development of two exemplary optical MEMS devices with the aim of volume production comparing their experiences to CMOS manufacturing. The transfer of a MEMS research prototype toward a mass producible product is further described in detail by Fitzgerald et al. [35,49] giving a plethora of hints and tips for commercial success. They highlight the differences between the initial research prototype created with an academic mindset and the requirements for volume production. They suggest to take an intermediate step and further elaborate the research prototype using a process called translational engineering to an advanced prototype, which is then ready to be transferred to volume production for commercialization.

MEMS devices that are currently attracting a lot of research interest are magnetoelectric (ME) sensors [50,51,52,53]. They allow sensing small magnetic fields and show promising results for biomagnetic and magnetic field-assisted medical applications [52,53,54,55,56]. Their concept is based on the coupling of a magnetostrictive and a piezoelectric functional layer in a mechanical resonator, which can be realized as a cantilever structure using MEMS technology [57,58,59,60,61,62]. Different read-out schemes are possible for such a device, for example exciting the resonator with a changing magnetic field of matching frequency [63,64]. In another read-out scheme, the resonator is excited with an electrical driving signal observing a magnetic field from changes in the resonator’s properties caused by the magnetostrictive layer (so called delta-E effect) [65,66,67,68].

In this work, we share our experiences with fabricating MEMS ME sensors in the context of a collaborative research project joining cutting-edge sensor technology research with advanced biomedical applications (Collaborative Research Center CRC 1261). Over the course of the research program, the demand for reliable and reproducible sensor fabrication arose. To serve this demand from the application fields in the scope of a central service subproject, we strongly profited from insights into a MEMS production environment. The central hypothesis is that even in the dynamics of a research endeavor, it is beneficial to adapt and apply tools and methods deduced from volume production. The key step is to change the perspective from fabricating devices to developing, maintaining and advancing the fabrication process. While the final goal still is the successful fabrication of devices, the change in perspective promotes focusing on the necessary actions to manage quality, risk and schedules, almost reducing the fabricated devices to side products. Methods, tools and the overall approach are presented and discussed using the fabrication of ME sensors as an illustrative example.

## 2. Materials and Methods

To structure the presentation and discussion of the methods and tools used in the suggested approach, they are assigned to one of the following three phases occurring during MEMS development: Efforts and investigations made before working on a particular technological implementation are grouped in situation analysis. Process integration describes the work of developing the particular technological solution, while execution comprises the tangible processing of material and fabrication of devices.

Please note that only in an ideal case, these phases are passed in sequence executing tools and methods in the order shown (which partially is chosen with respect to optimizing the reading experience). The intricate interdependency between device design and fabrication process often makes iterations within and between phases necessary.

### 2.1. Situation Analysis

The tools and methods grouped in situation analysis aim to obtain clarity on the object of study and serve to find and confine boundary conditions and circumstances. A technical problem is posed, which is to be solved by the MEMS technology to develop. Any prior knowledge should be gathered, and the state of the art should be investigated to best define the starting point.

#### 2.1.1. Prior Results

In the chosen example, the target is used to reliably and reproducibly fabricate ME sensors for application research. The bulk micromachined delta-E effect ME sensor reported by Zabel et al. [59,66] was chosen due to its promising performance and its favorability with regard to system integration aspects. These sensors consist of a 3 mm by 1 mm single-side clamped cantilever made from a 50 µm thick poly-crystalline silicon (poly-Si) layer. A 2 µm thick piezoelectric AlN layer deposited and structured on the front side connects electrical and mechanical domains. The magnetostrictive ${\left({\mathrm{Fe}}_{90}{\mathrm{Co}}_{10}\right)}_{78}{\mathrm{Si}}_{12}B_{10}$ multilayer with a total thickness of 2 µm is deposited and structured on the back side of the wet chemically released poly-Si layer to connect magnetic and mechanical domains. The cantilever geometry is defined by a final deep reactive ion etching (DRIE) step using the Bosch process before the devices are singulated using saw dicing and assembled (compare also Section 2.2.1 and Appendix A). Being the result of a scientific venture, this MEMS device and fabrication process qualify as a research prototype as described by Fitzgerald et al. [49].

#### 2.1.2. Preliminary Specifications

In an industrial context, a precise and thorough specification is a crucial prerequisite for producing MEMS devices. It translates the requirements from the application or customer to requirements on the MEMS device and further to specifications of the device and the fabrication process. Specifications define all necessary properties and features and thus determine quality targets and provide limitations by which the fabricated devices are accepted (in-spec) or rejected (out-of-spec). Fixing specifications is necessary when starting the transfer to production. However, it is good practice to start the specification process as early as possible [37,46,47].

The documented specifications serve as a guideline throughout the development process highlighting critical parameters. They further ease communication, as they capture the expectations of all stakeholders. However, it is important to understand that the initial specifications recorded at this stage are subject to change with progressing development, e.g., when process limitations come to light. To distinguish them from the device and process features guaranteed by frozen specifications used in production, it is advisable to denominate them as *preliminary specifications*. It has been observed for large engineering projects that an early commitment to premature specifications in an uncertain situation can have negative consequences for the overall project progress [69].

Table 1 shows the preliminary specifications at the beginning of the development of the ME sensor used as an example in this work. They result from target applications, underlying the working principle and circuitry intended for driving and read-out. It is quite common to start with a manageable list focusing on the most important items and making the limits as wide as possible, which are then continuously amended and confined over the development process. The role of the customer is important in this context; in the case of the presented example, it is filled by the projects studying the applications of the developed ME sensors.

#### 2.1.3. Cycle Time Analysis

Cycle time analysis is a method used in the semiconductor industry to minimize lead times, reduce work-in-process material, increase equipment usage and maximize yield to optimize the overall economic performance of a fab [70,71]. Strategic decisions are informed by models, simulations, and the analysis of big data sets generated during operation of the fab under study.

Inspired by these activities, a cycle time analysis of a fabrication flow under investigation can be utilized to learn about limitations and bottlenecks, even in a research attempt. The effort of the analysis is cut drastically by reducing it to the decomposition of the fabrication flow to the theoretical cycle times of its single process steps, relying—where necessary—on estimations rather than precise measurements. The theoretical cycle time is the bare minimum time necessary for completing a process step under ideal conditions, in particular neglecting influences from the availability of operators and equipment. To capture a potential scaling with batch size, e.g., from the parallel processing of multiple wafers in the same tool, the cycle time of each process step is modeled to consist of a fixed time per run $w_{fix}$ and a variable time scaling linearly with the batch size. When $w_{var}$ represents the variable time per material unit and the batch size *n* is the number of material units in the batch, the theoretical cycle time of the *i*-th step $CT_{theo,i}$ derives to

(1) $$CT_{theo,i}=w_{fix,i}+n\;w_{var,i}.$$

While in a production line, the occurrence of a bottleneck is influenced by many more factors (e.g., operator and tool availability, capacity utilization, other products), the theoretical cycle time of a process step is a good indicator for a potential bottleneck in a research-focused environment, given its limited redundancy and interoperability. If additional development is necessary to establish a process step, cycle times can be utilized to estimate the development effort based on the expected number of iterations.

**Figure 1:**
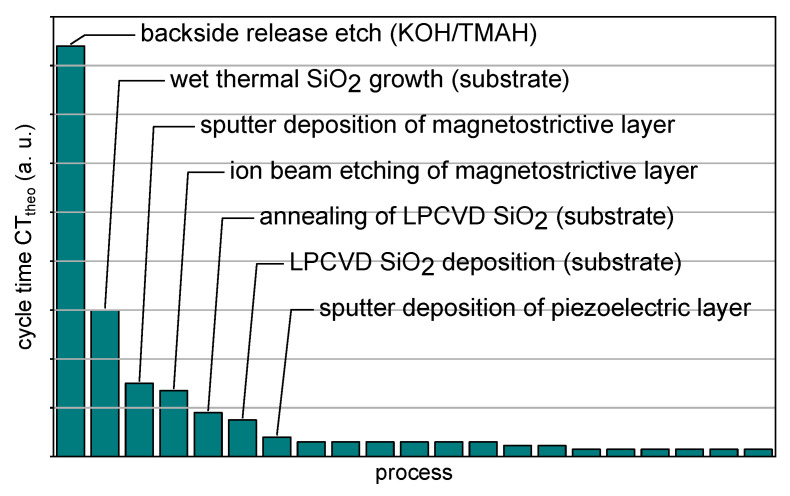
Cycle time analysis of the initially sketched ME sensor fabrication flow. The estimated theoretical cycle times for fabricating a single 50 mm square substrate are plotted for the processes in descending order to reveal potential bottlenecks.

In Figure 1, the estimated theoretical process cycle times for a single material unit (n=1) on the fabrication flow outlined above are plotted in descending order. The seven processes with the longest cycle times are identified and labeled. The wet chemical etching to release the poly-Si layer from the bulk substrate has by far the highest cycle time. Oxide growth, deposition and annealing to prepare the poly-Si substrates are processes with high cycle times as well. However, these processes allow large batch sizes, such that increasing the batch size under consideration would mitigate their impact. In contrast, the sputter deposition of magnetostrictive and piezoelectric functional layers as well as the dry etching process to structure the magnetostrictive layer are single-wafer processes. Their impact is independent of the batch size, and the analysis motivates keeping them in mind.

Cycle time analysis can further be used to learn about the overall time required to fabricate devices. While a research endeavor is not under the same economic pressure as industry, there are still timelines and project plans to be met. Additional information is necessary to convert the theoretical cycle times into a real time frame, e.g., the common wait time in front of process steps, availability of tools and workforce, working time per day and workdays as well as planned breaks. Additional work packages for process development have to be taken into account, where necessary, and the overall uncertainty resulting from the assumptions made must not be neglected. While demonstrated here as part of the situation analysis to educate the starting point, it is good practice to update and reevaluate the analysis with the fabrication flow resulting from the upcoming process integration efforts.

#### 2.1.4. Boundary Conditions

Before starting the process integration considerations, it is advisable to identify further boundary conditions for the development of the MEMS device and fabrication process. Technical limitations may for example result from the available processes, equipment, and their compatibility, indicating potential gaps. The following boundary conditions have been identified for the ME sensor example:

Two sites are available for processing: the cleanroom of the Kiel Nanolaboratory at Kiel University operating mostly on 150 mm-wafers and the cleanroom of the Fraunhofer Institute for Silicon Technology (ISIT) in Itzehoe operating on 200 mm-wafers. While the Kiel Nanolaboratory offers quick access to cutting-edge material development, the ISIT facilities are performing research and development in a production-relevant environment.

The magnetostrictive layer system is actively being researched within the CRC 1261. The process flow needs to be able to integrate new findings, such as exchange biased multilayer systems [68]. Depending on the details of the deposition process, it can be limited to the use of 50 mm square substrates. The deposition and structuring of these material systems are not available in the ISIT cleanroom.

### 2.2. Process Integration

The task of process integration—the art of combining single processes to create something greater—is the development of the particular technological solution to fulfill the specifications while adhering to the boundary conditions found in situation analysis. A peculiarity of MEMS development is that at this stage, the device design is not fixed. Due to the entanglement of design and process, it is necessary to be able to adapt the design to constraints and opportunities found during the implementation of the fabrication process. The preliminary specifications serve to guide these decisions, as they indicate where changes are tolerable and which limits must not be exceeded. Especially in a research context, the device design can change significantly during process integration, as fabrication processes are pushed to their limits.

Completing the development of the technical solution, process integration will yield a number of documents allowing to self-check the completion of the various tasks, as detailed in the following. The description of device design and process flow are supplemented by design rules, cross-sections, mask alignment strategy and layout data. In addition, the test concept is fixed in the control plan with test structures incorporated in the layout. Risks in the device design and the process flow are identified and documented in failure mode and effects analysis (FMEA). While it is strongly advised to complete this set of tasks before starting the execution, it is at least as important to update them to changes found necessary at a later point.

#### 2.2.1. Design and Process

**Figure 2:**
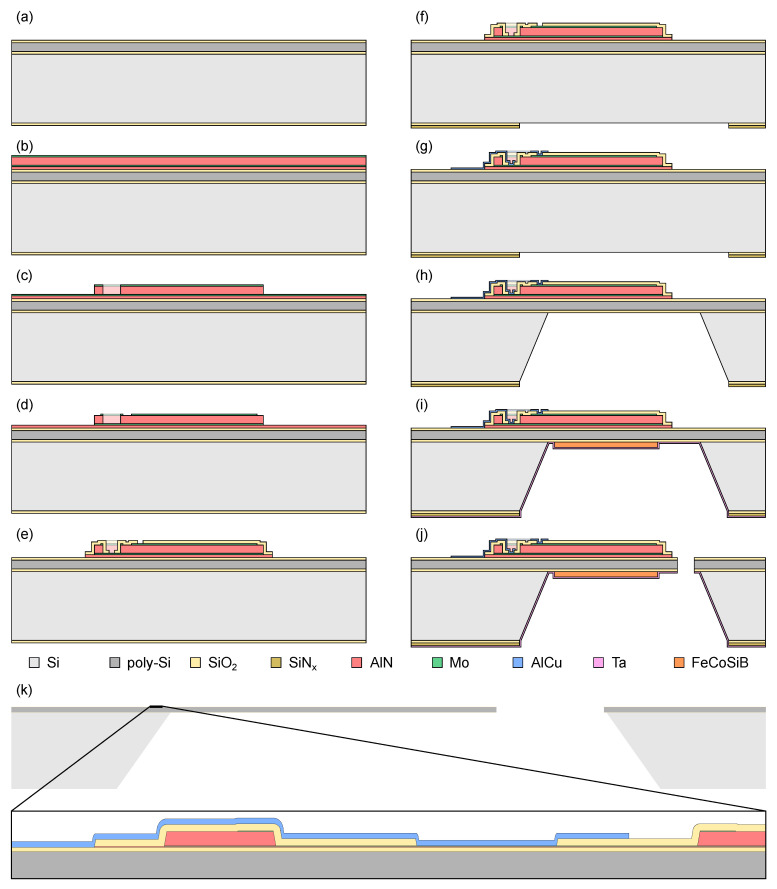
Schematic cross-sections illustrating the process flow developed to fabricate ME sensors: (**a**) polished Si substrate with poly-Si layer, (**b**) deposition of piezoelectric layer stack, (**c**) structuring of the AlN layer, (**d**) structuring of the electrodes, (**e**) deposition and structuring of isolation layer, (**f**) preparation of hard mask for back side release, (**g**) deposition and structuring of contact metal, (**h**) back side release of poly-Si layer, (**i**) deposition and structuring of magnetostrictive layer, (**j**) definition of cantilever structure etching the poly-Si layer. For comparison, (**k**) shows a simulated cross-section of a 7 mm die in the final state with the inset focusing on the bottom electrode contact. Colors refer to the different materials, as indicated in the legend.

The two most important items of process integration are the design of the device and the process flow for fabrication. While the device design is best described in a sketch, the process flow is documented usually in form of a spreadsheet listing all process steps, their parameters and the equipment used. Both meet in the drawing of cross-sections, which are invaluable in use for communication and documentation. While the device design can be seen from the final cross-section, the process flow can be followed by the sequence of intermediate cross-sections. Figure 2a–j exemplarily show a selection of schematic cross-sections for the process flow developed to fabricate ME sensors. Even if only a limited number of intermediate steps are shown here, it is good practice to invest the effort to draw detailed small-stepped cross-sections (including, e.g., resist masks). It would not be the first time that an incompatibility of a process step with some layer opened locally or missing access to alignment marks is only found when drawing cross-sections of the specific steps. To be able to interpret cross-sections, an idea of the third dimension has to be present. This can be achieved either from a sketch of the device or the plan view layout.

The physical devices will later be fabricated from the process flow and the mask data containing the plan view layout. As this information sufficiently describes the fabrication and its outcome, it can as well be used to simulate the process in advance. Sophisticated software tools have been developed to help with this task, such as those for example demonstrated by Bieniek et al. [48]. A simpler solution available under public license (GPL) is based on the popular layout tool *KLayout* [72]. The extension *xsection* [73] allows simulating cross-sectional views from the plan view layout and a list of instructions derived from the process steps compiled in a script. An exemplary cross-section of the final state created using this work flow is shown in Figure 2k. As immediately visible, the true scale complicates capturing all relevant details in one view compared to the manually sketched cross-section of Figure 2j. However, once set up, such a work flow allows easily generating cross-sections of arbitrary positions in the layout, which are best explored in an interactive way. It is a valuable tool to check the layout for errors, indicate challenging aspects of the process flow and facilitate communication within and beyond the group of process experts. As the layout has to be drawn inevitably to generate the mask data, the great benefit for little effort provide motivation to start drawing the layout (at least in parts) early on during process integration.

To assess the interdependency of design and process in a systematic way, design rules are a concept commonly applied to provide guidance during design and layout work. They are rules derived from the process flow, which are necessary to ensure a successful fabrication within the given limits. Thus, the endless design space of a technology is confined to its realizable implementations. The rules are usually formulated in the language of the plan view layout based on the different lithographic layers to define the design, e.g., imposing minimum and maximum widths of certain structures or two layers excluding each other. The effectiveness of design rules for production becomes apparent from a closer look at the foundry business model. Established from microelectronics manufacturing, foundries provide process technology and fabricate devices based on customer-specific designs [35,47]. This puts the interface of foundry and customer right at the interplay of design and process. To ensure a smooth operation (technologically as well as in terms of business relationship), foundries provide well-documented design rules as part of their process design kits. Automated design rule checks (DRC) are implemented, examining the layouts created by the designers. The practice of formulating and documenting design rules for a process flow should be adopted, even in research-focused process integration. It helps avoid disconnects between design and process development. Automated DRC can be beneficial for large and complex designs, but their implementation is a non-negligible effort.

Another task adding to the due diligence of process integration is the development of a mask alignment strategy. The fabrication of MEMS devices relies on the precise overlay of several structuring layers. The peculiarities of a process flow dictate which marks created in preceding layers are visible at which stage and can be used for alignment. A careful consideration and documentation avoids complications during execution.

In MEMS development, finding the right process flow to fabricate a particular device design does not follow a straight relation. The solution space is wide open, yielding many different technological implementations. Some of them are better, while others are less suited, but usually coming with different sets of advantages and disadvantages. The criteria for judging process flows depend not only on technological aspects but are in many cases influenced by additional boundary conditions. In the following, decisions made for the process flow to fabricate ME sensors are presented exemplarily. A detailed description of the process flow is given in Appendix A.

The need for reliable and reproducible sensor fabrication within the CRC 1261 and the unique situation of having access to two complementary cleanroom facilities led to the key consideration to split the process flow in a cross-institutional manner. Up to the magnetostrictive layer deposition, the devices are processed in the form of 200 mm-wafers in the ISIT cleanroom, benefiting from processing in a production-relevant environment. These wafers are cut down to 50 mm square substrates for magnetostrictive layer deposition and structuring at the Kiel Nanolaboratory, benefiting from the latest developments in this field. Due to the substrate size, the remaining process steps can only be performed in the Kiel Nanolaboratory. The fabrication throughput up to the magnetostrictive layer deposition is substantially increased using processes on 200 mm wafers. In particular, the back side release etch identified as a potential bottleneck process in the cycle time analysis is sped up by processing 200 mm wafers, yielding 8 of the 50 mm square substrates per wafer. A similar effect is anticipated for the other processes, increasing the weight of magnetostrictive layer deposition and structuring on the overall cycle time.

Compared to Zabel et al. [59], the process flow is simplified using Mo for both the top and bottom electrode. This allows structuring both electrodes in one etching step, reducing the number of overall layers. Contrarily, two additional layers are introduced to allow contact wiring in a dedicated metal layer to optimize interfacing by wire bonding and reduce parasitic capacitance compared to contact lines made from the electrodes (compare the preliminary specifications in Table 1). Due to the process availability constraints resulting from the decision to split the process flow in a cross-institutional manner, the opening of the back side hard mask for the release etch had to be postponed until after removing the AlN seed layer in TMAH, as the exposed Si would lead to contamination. Similar to the previously reported process flow [59], the magnetostrictive layer is added to the devices in the latest possible step due to its sensibility to temperatures above 250 °C [74] limiting further processing.

The chemical compatibility and thermal budget mentioned above are only a few examples for technological constraints, which have to be managed carefully in the overall process integration effort. Often, the many details are only learned from experience, so discussing a process flow proposal with experienced engineers and scientists becomes mandatory to reduce processing and development risks. In an effort to overcome this limiting factor, an extensive software environment has been developed [38,47].

#### 2.2.2. Test Concept and Control Plan

Quality management is a highly elaborated part of production. In contrast to research operated in academia, where success and failure are both viable outcomes of an experiment increasing our knowledge, the commercial pressure in industry motivates extensive efforts in production to maintain quality at a high level. MEMS research can highly benefit from introducing quality management fundamentals learned from a production environment. The key point to quality in production is testing, i.e., measuring the result of fabrication processes to ensure they stay within the specified limits [35,37,49]. Depending on their position within the process flow, measurements can be grouped to inline, end-of-line and product testing.

Inline measurements are integrated into the process flow, allowing testing the outcome of processes close to their execution. The quick feedback allows reacting to non-conformal results, e.g., resolving the situation by a rework procedure or rejecting the material to avoid wasting resources on further processing. Some parameters can only be measured inline, as they might not be accessible in the final state. End-of-line measurements are executed on dies after the last processing step. At this stage, the devices have almost their complete functionality, allowing the characterization of a multitude of properties at once. Being still grouped by the substrates used for fabrication optimizes handling and automation. Defective parts identified at this stage can be excluded from assembly and packaging. Product tests are executed on the final device after assembly, packaging and any postprocessing steps. They allow accessing the performance in application-related setups and environments, which after all are the most significant for the later usage of the product. The groups of tests do not exclude each other and are usually applied in a well-designed combination. While the product tests are usually determined by application and reliability requirements, the complex semiconductor fabrication processes motivate early inline tests. This is described in the literature as trade-off between timeliness and quality of information [35,49].

A variety of measuring methods is applied for MEMS technology, with applicability, limitations and the need for standardization discussed in the literature [75,76,77]. The main limitation for inline testing is the applicability of the measurement method on the substrate size used for processing and avoidance of contamination conflicting with further processing. A particular craft in MEMS testing adopted from the semiconductor industry is the design of test structures to characterize specific properties, which is often referred to as process control monitors (PCMs) [35,75,76]. These structures—together with their measurement method—are tailored to be sensitive to target parameters while preferably insensitive to other fluctuations and usually are developed together with the fabrication process. The use of test structures probed electrically is especially popular as they can be characterized collectively in an end-of-line test using highly automated wafer probing equipment and are founded on experiences developed over the last few decades. Another test method inherent to semiconductor manufacturing is the search and documentation of defects, e.g., by optical inspection. Sophisticated frameworks have been developed to automatize and speed up this process, as in contrast to the aforementioned electrical characterization confined in a single end-of-line process step, the check and classification of defects usually is executed repeatedly at different stages in the process flow.

**Figure 3:**
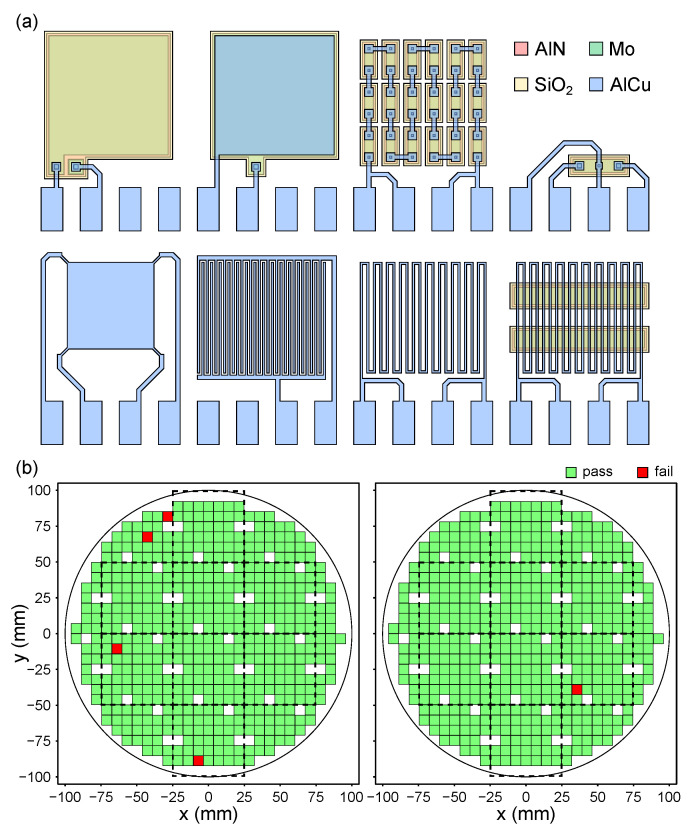
(**a**) Plan view layout of exemplary test structures employed in the ME sensor fabrication process; see Appendix B for a detailed description. (**b**) Characterization results of the intermediate 100% measurement of electrical structures on 200 mm wafers to educate further processing on 50 mm square substrates indicated by dashed lines.

In addition to several inline measurements such as optical inspections, wafer bow and thickness measurements for deposited layers, a set of 42 test structures has been designed for the ME sensor process flow to be characterized electrically in an automated measurement process. While resembling the manner of an end-of-line test, this characterization is placed after finishing the metal layer and before the back side release. At this stage, the electrical structure of the devices is finished, allowing an early characterization, while the bulk 200 mm wafer substrates enable an automated handling in the employed equipment. Figure 3a exemplarily shows 8 of the used test structures, and a detailed description is given in Appendix B.

As the test structures are designed using the same technology as the devices, the results of several test structures have to be consolidated for a meaningful conclusion. Using the given example, the finger, meander and sheet resistance structures in the contact metal layer need to be considered first to make sure the layer used for contacting behaves as expected. Next, the properties of the isolation layer can be addressed using the plate capacitor structure, together with the ability to contact the electrodes through contact vias by the respective test structures. The properties of the electrodes can be monitored using structures similar to the metal layer, which is not shown in Figure 3a. Only if all these structures show the expected behavior can the properties of the AlN layer unambiguously be interpreted from the results of the corresponding plate capacitor test structure.

In addition to the dedicated test structures, an electrical 100% measurement on the later ME sensor devices is executed to identify defective parts. The results for two wafers are exemplarily shown in Figure 3b, overlaying the positions of the 50 mm square substrates used for the magnetostrictive layer process module. This allows evaluating the quality of the fabricated intermediate parts to optimize the use of resources in further processing. The final ME sensor devices after assembly and packaging are characterized extensively for usage in research applications within the CRC 1261.

Measurements during fabrication need to be treated like any other process step and included in the process flow. As there is a significant amount of additional information to be considered for testing (e.g., test structures, expected values and limits, potential causes and solutions), the documentation in a separate control plan is advisable. To decide on the amount of measurements to be incorporated during the research and development phase, several aspects have to be brought in balance. On the one hand, additional measurements allow accessing the quality of the fabrication process and devices already during processing and open possibilities to react on unforeseen situations. On the other hand, they can significantly prolong the overall cycle time.

In contrast to a commercial production, MEMS research and development is encouraged to make extensive use of measurements and testing, acting out a scientific mindset within reasonable limits. Pushing the boundaries of the known, unforeseen results are likely to occur and the density of data points is an important factor for the interpretability of results. From a manufacturing perspective, the measurements performed during initial research and development not only serve to find defects and non-conformal process results: they are also used to establish the measurement methods and record a baseline of conformal process results. Nevertheless, the efforts caused and resources bound by the measurement steps should be monitored critically, and a reduction of measurements with increasing experience with the new process flow is advised. Automated measurement should be employed where possible. However, due to the dynamics of a research-focused development, the gain has to be balanced against the effort needed for setup.

#### 2.2.3. Failure Mode and Effects Analysis

Another important pillar of quality management actively pursued in production is the usage of risk management methods such as failure mode and effects analysis (FMEA) [35,78,79,80,81]. Aiming to increase the overall quality, safety and reliability of product and fabrication, FMEA works by systematically identifying potential failure modes, their causes and effects. These are then evaluated by their severity, occurrence and detectability, and actions are deduced to mitigate the associated risks. While best performed in teams with complementary expertise [80], an FMEA is regularly revised and dynamically updated as new failure modes are identified.

Based on recommendations of the automotive industry [82], design (D-FMEA) and process FMEA (P-FMEA) can be distinguished. D-FMEA addresses risks associated with the device design, while P-FMEA covers fabrication risks. Links between the two can be observed in the shape of failure effects and failure modes identified in P-FMEA becoming failure modes and failure causes in D-FMEA, respectively [82]. While this generally applies as well in MEMS development, there is an additional link of D- and P-FMEA becoming apparent. The entanglement of design and process leads to mitigation measures of failure modes identified in P-FMEA often involving changes to the design, especially during the development phase. Despite this entanglement, the differentiation of D- and P-FMEA stays advisable also in the MEMS case. The perspectives used to systematically reveal potential failure modes are complementary in approach. In D-FMEA, the device is broken down into its sub-parts and their functions, while in P-FMEA, the process flow is examined step by step.

Table 2 and Table 3 exemplarily show excerpts of the D- and P-FMEA performed for the developed ME sensor. The guidelines used in the automotive industry [82] are adapted to fit MEMS development, including the evaluation criteria for severity, occurrence and detection (see Appendix C). Further simplifications have been made to best suit the research nature and needs of this project, such as trimming the tracking of and reevaluation after implementation of mitigation measures. It is noted that in a research context, the incompleteness of the preliminary specifications often obfuscates the evaluation of the failure modes. The evaluation nevertheless is a valuable tool as it guides thinking to the three main criteria and helps addressing the various risks with appropriate priority.

Performing FMEA comes with a significant effort, especially when leveraging the expertise and experience of a large and diverse team, raising the question of appropriateness for a research endeavor. The latest point in time to perform FMEA in product development is before the freeze of device design and process flow. Care has to be taken to stay focused and not get lost in hypothetical thought experiments and the formalisms of FMEA templates. As stated above, the search for the best design and process implementations inevitably involves exchange with experienced experts. FMEA can be seen as a chance to put this into a systematic frame guiding through the development process to minimize the risk of missed issues. Using FMEA to frame process integration reduces any additional efforts and ensures benefiting from its positive effects. FMEA is therefore best started early in parallel to the other process integration tasks. At this stage, device design and process flow still have the highest flexibility to implement changes. P-FMEA can be seen as part of the criteria judging different process options, while D-FMEA can educate device design decisions. When carefully adopted to the situation, the usage of FMEA is highly recommendable even in a research project.

Being a useful frame for process integration, FMEA can have a significant effect on the other tasks described above. The systematic questioning of device design and process flow can lead to changes to one or the other in order to mitigate risks associated with potential failure modes. This often triggers a cascade of updates to the above-mentioned documents up to the FMEA itself (compare Section 2.3.4), turning FMEA to the driver of iterations in process integration. A particular link also exists to the testing concept and control plan. Risks identified in design or process evaluated below the threshold of changes often motivate the introduction of additional measurements and dedicated test structures for monitoring.

### 2.3. Execution

Execution describes the phase of physically performing the process steps in sequence according to the process flow and using masks generated from the layout data. This is the core of production and taken to its best in industry, where a process flow is executed very many times in volume production. Driven by the high cost faced in semiconductor processing and challenged by competitive lead time promises, downtime and idle time are the main adversaries of production. While this is not quite transferable to research-focused MEMS development, there are interesting aspects to be learned as well.

#### 2.3.1. Production Control

The production control in any larger semiconductor fabrication plant (fab) is nowadays accomplished by means of a manufacturing execution system (MES), enabling planning, scheduling, quality control and performance analysis [83,84]. The MES is aware of every equipment and its status, all single processes as well as the process flows of all products and every material running and its position in its flow. The history of lots and equipment are logged for traceability and proof. Measurement data are captured to not only apply quality limits to route the current material but also track and study the process performance from run to run.

While the same obligations regarding traceability and documentation exist in a research context, the effort of maintaining an MES for a research-only facility usually is seen to exceed the benefits. Instead, solutions based on run sheets and equipment logbooks are widely used. The run sheets travel with the lots containing a list of all process steps with necessary process parameters and sign-off to track their execution. The equipment log books trace the materials processed as well as maintenance and qualification performed. To allow the interpretation of results and the analysis of any deviations, the implemented solution has to allow analyzing the sequence of process steps and parameters seen by a particular material and the condition of the equipment used for processing including recent events and processing history.

In the example of the developed ME sensor process flow, the processing taking place at the ISIT facilities is controlled and tracked by the obligatory MES. For the process steps performed in the Kiel Nanolaboratory, a run sheet and equipment logbook system is used. Due to the extent, value and intimacy of the information processed in an MES, a usage beyond corporate boundaries is prohibitive.

#### 2.3.2. Process Control

Maintaining process stability is an art in and of itself and a critical necessity to ensure consistent fabrication within the specification limits. Volume production benefits from the huge number of lots processed (ideally without longer interruptions) generating dense data. Run-to-run control is usually employed based on statistical (SPC) and advanced process control (APC) methods [85,86,87]. As stated in Section 2.2.2, dedicated test structures as PCMs play an important role.

In research and development, material numbers are limited, and breaks between the runs of specific process steps can be long. A question to be answered for every process step, e.g., in context of a P-FMEA (compare Section 2.2.3) is how the conformity of the process result is ensured and/or deviations are caught. Recommendable measures include process qualification executed on monitor wafers prior to running hot material to catch any abnormal tool and process conditions and additional measurements on the processed material.

Not only are the material numbers per project limited in a research context, the projects often aim to explore the unknown by trying new processes and process sequences. This makes it even more important to mitigate the sparsity of data generated on fabrication steps by close coordination between different projects. Data on common process steps might be merged to pass a critical limit. Potential differences resulting from the process history and layout of material from different projects have to be considered carefully. To recall, the concept of PCM allocates test structures to particular process steps rather than a process flow or product, thus promoting a common use.

#### 2.3.3. Material Flow

Another way to mitigate risks and optimize the quality and schedule of fabrication is manipulating the flow of material [35,49]. It plays an important role during the development of fabrication processes, where the final process flow is not fixed yet. The three elementary functions to manipulate material flow are splitting the material of a lot into multiple sublots processed independently, holding a lot to synchronize different timelines and merging material from multiple lots into a single lot to subsequently be processed together. A detailed description of risk mitigation practices manipulating material flow is given by Fitzgerald et al. [35].

A best practice used at high-risk process steps is the use of so-called *send-ahead* material. A small number of wafers is split from the main lot and processed as planned up to the point where the outcome of the particular process step can be judged. If the result is positive, the main material is processed to catch up with the send-ahead material subsequently being merged. If not, the process is adjusted and the procedure is repeated with another send-ahead material. In contrast to monitor wafers used for the qualification of single process steps, the processing of send-ahead wafers continues along with the main material potentially yielding devices.

Short-loop material is used to speed up process development and reduce the necessary investment. A split lot skipping parts of the process flow allows a faster supply of material to a process step with pending development. The short-loop material is used and consumed for the initial development to start with a higher level of knowledge once it is the turn of the full-loop material.

Tuning the material flow is the most illustrative support for the hypothesized benefit of changing the perspective to focus on developing, maintaining and advancing the process flow rather than processing of single wafers. A continuous flow of material—as seen in volume production—is the most effective way to mitigate the risk of critical delays or shortages in delivery. Having material at different stages of the process flow can serve as fallback when a critical situation is faced, mitigating the overall effect. At the same time, this continuous flow of material acts as an abundant pool for development targeting to further enhance process and device performance.

However, in research and development, the implied cost makes this an unrealistic resolution. Cost and benefit have to be balanced as part of risk management, often resulting in holding back material only at particular stages in the process flow. In the example of the developed ME sensors, one of these points is after the deposition of the piezoelectric layer. Still being before the first lithography layer, material at this stage is insensible to revisions of the layout while already having accumulated significant processing. The high cycle times regarding the ${\mathrm{SiO}}_{2}$ layers of the substrates make preprocessing material in larger batches up to this point additionally favorable.

#### 2.3.4. Change Management

Due to the high requirements on semiconductor processes and their complex interdependencies, changes to design or process—appearing small on first sight—can turn out to have tremendous effects. To protect production against imprudent consequences, changes are managed in a change request system [47,88]. A change request, formalizing the proposed changes and analyzing its consequences, is distributed to systematically capture all relevant factors and risks. All relevant stakeholders have to agree for a change to come into effect, which if necessary is accompanied by an additional conditionality. The rigidity of this procedure is necessary, as design and process are frozen for product qualification and yield management.

While a full-grown change request system is disproportionate for a research endeavor relying on scientific freedom and creativity to pursue innovation, it can still teach us a lesson in deductive reasoning and serenity. It is a good exercise to put the idea for a change down in a document similar to a change request. The transfer to writing can act as a first self-examination of the implications and effects of a change. Once formulated, it eases communication with peers to gather additional opinions and advices.

**Figure 4:**
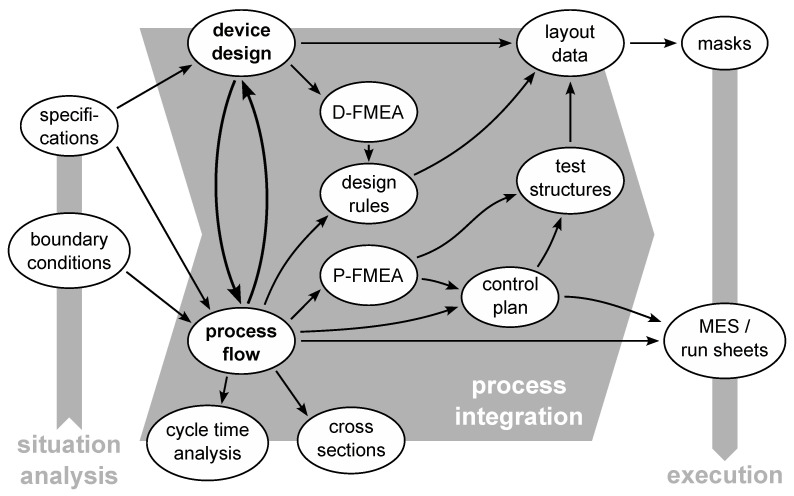
Illustration of the dependencies between the tools and methods and their outcome described in this work to establish a workflow for changes.

Considering changes implies reconsidering parts of process integration and potentially triggering cascades in updating documents, which are similar to the iterations gone through to mature device design and process flow before the start of execution (compare Section 2.2). To illustrate the workflow and aid in performing updates related to changes, the dependencies between the different tasks and their outcome is shown in Figure 4. If one of the items is changed, the dependent items have to be considered for associated changes as well.

An illustrative example is the additional SiN layer used in the developed ME sensor fabrication for the back side release hard mask. In the first P-FMEA, the ${\mathrm{SiO}}_{2}$ layer initially intended as a hard mask has raised concerns regarding insufficient thickness and/or selectivity in the etching process. To mitigate the identified risk, the development was pulled in using short-loop material with results indicating the need for an additional SiN layer. The process flow was the first item affected by this change (see Figure 4). Following the dependencies, the device design was updated for the additional layer, but no effect on layout or D-FMEA has been found. Completing the dependencies sketched for the process flow, the cross-sections and cycle time analysis were updated, while the design rules stayed unaltered. After revising the P-FMEA, the control plan was amended for an additional measurement of deposited SiN thickness without the need for a dedicated test structure, and the changes were implemented in the MES, completing the envisioned change.

## 3. Discussion

Three phases are chosen in this work to structure the presentation of different tools and methods used during MEMS development: situation analysis, process integration and execution. In an ideal case, running through these phases only once—following the dependency pattern shown in Figure 4—could be sufficient to arrive at the solution of the development task. Due to the intricate interdependencies in MEMS design and fabrication, this is usually not the case. Despite the reported efforts [36,38,42,43,44,45,46], MEMS design is still dominated by an expertise-based approach. As highlighted by Sagoo et al. [46], this leads to a number of iterations commonly experienced during the development of an MEMS device and its fabrication process. From an industrial perspective, these loops are the main cost and time factors in product development. In a research context, they are unavoidable implications of the learning process provoked by pushing the limits of what is possible. In both cases, the aim is to reduce their number, keep them short and run them in a controlled and cost-effective way.

Ideas for necessary or favorable changes can be sparked anytime in the development process: for example, when attempting a critical process step for the first time or analyzing the results of the first finished devices. As significant cost is associated with processing material, it is worthwhile trying to evaluate and implement as many of these changes during initial process integration before starting the execution. This is the first-time-right fabrication objective pursued in design for manufacturing and strongly motivates the use of D- and P-FMEA methods. To not lose clarity of the dependencies shown in Figure 4, the possible iteration loops have been omitted. They can be considered leading to separate instances of the sketched workflow, restarting at the first item affected by the change.

While giving a rough outline on MEMS development, the chosen three-phase structure is not intended to compete with the thorough product development schemes of Ortloff et al. [47] and Fitzgerald et al. [35,49], covering far more than only design and process development. Being the result of a scientific venture, the ME sensor device and fabrication process reported by Zabel et al. [59,66] qualifies as a research prototype following the classification scheme described by Fitzgerald et al. Accordingly, an intermediate step would be necessary before initiating the transfer to production [49]. A similar classification scheme can be found in the automotive industry distinguishing parts fabricated with different maturity levels during the product development process. In between the initial demonstrator (A-sample) and the first qualification samples (C-sample), B-samples describe parts produced at an intermediate level with production-relevant processes, exhibiting a high similarity to the advanced prototype in MEMS development. Considering the ongoing development with respect to the magnetostrictive layer in the ME sensor example, the presented state only partly fulfills the criteria for the advanced prototype or B-samples. Pushing most aspects of the technology toward the advanced prototype level facilitates the development and is a significant step toward reliable and reproducible device fabrication.

Both the works by Ortloff et al. [47] and Fitzgerald et al. [35,49] have in common that the commercial success of the developed MEMS product is in focus. This is in contrast to the goals of the research project this work draws the presented experiences from, where scientific progress and pioneering are in focus. Both have in common that resource budgets and timelines have to be met. However, while for commercial success, technology needs to be pushed to the point where it can be transferred to volume production, in a research context, technology should be pushed to its physical limits to facilitate discoveries.

This raises the question of whether the demonstrated approach adopting tools and methods from production to a research context is appropriate. In particular, the additional efforts have to be judged with respect to the gained benefits. From our experience, the approach of focusing on developing, maintaining and advancing the fabrication process is highly advantageous, as seen in the demonstrated example. The additional effort invested in the beginning to mitigate technological risks has led to a smoother execution and thereby reducing later costs of potential material intensive iterations. The overhead of continuously applying the methods is manageable once they have become part of daily operations. In fact, they to a large extent replace tasks and documents which are necessary anyway, and the systematic framework facilitates the execution.

While the chosen tools and methods are inspired by the practices seen in volume production, where they are executed to their best, they are not exclusively immanent to it. They have been developed to answer the specifics of MEMS and semiconductor fabrication relying on highly specialized processes. The high commercial pressure in industrial production has fueled their advancement, becoming an active research field of its own. While it can only be beneficial to learn from the acquired knowledge, care has to be taken to not lose sight of the peculiarities of a research endeavor. Attempting to convert a research project into a production line will likely fail. Careful adoption is necessary to balance the efforts versus gained benefits. In addition, the decisions and details can differ between projects.

Going one step further, the question arises as to whether the investigation leading to the initial research prototype could benefit from the proposed approach as well. Working on an open research question, the developed solution usually is not known at the beginning. Care has to be taken to not over confine the solution space, risking to rule out valid and valuable solutions. However, using a workflow similar to the above suggestion can also allow focusing efforts on a specific matter and exploiting synergetic effects to reduce the overall cost. As MEMS fabrication usually consists of a large number of subsequent process steps building upon each other, a considerable portion often is of less scientific interest. In addition, the complexity and interdependency make the outcome of a process under investigation highly sensitive to the performance of all preceding steps. These preceding steps, which are often seen as a mundane necessity to access the relevant points, can benefit strongly from the above tools and methods or the use of standardized semi-finished parts. Such an approach can speed up development by decreasing turn-around times to obtain experimental results. At the same time, it improves the performance and reliability of the fabricated devices. When applied regularly for multiple projects and devices, synergetic effects can be fostered to further decrease the overall effort required, eventually leading to a modularization of the available technology.

From a production perspective, the approach applied to the example leads to the question of whether different parts of a process flow can be at different maturity levels. To increase maturity for production, the next steps would include specification, design and process freeze followed by product qualification efforts. Requirements for this step are—among others—completed process development, stable processes, quality control and risk management implementation. In the example of the ME sensor process flow, these could be met for the first part of the process flow on 200 mm wafers. The overall fabrication could benefit from increased stability and decreased cycle times for these process steps. Examples for different maturity levels in process flows can be found considering the initial substrate. The fabrication of the 200 mm wafer itself is not considered to be part of the process flow, but it is practiced in high volume production by an external supplier.

However, this example also indicates the limitations of maintaining parts of a process flow on different maturity levels. If during development changes to the substrate become necessary, this would affect the running production (or imply the move to a different product or supplier). A similar situation could arise within the ME sensor process flow. Development in the later steps can require changes in the first steps. Depending on the necessary changes, effort invested in pushing the maturity of the first part of the process flow may have been in vain. Due to the high investments involved when qualifying processes for production, maintaining parts of process flows at different maturity levels has to be considered carefully. In the ME sensor example, based on the uncertainties of ongoing process development activities and the research-focused objective, pushing parts of the process flow to a production level while development is still ongoing on later parts is not an option. Nevertheless, efforts advancing the maturity of parts of the process flow, as would also be necessary for preparing it for production, can still be worth investing to partly leverage the benefits stated above. Regardless, to avoid confusion when discussing maturity levels, it has to be kept in mind that the overall maturity of a process flow can only be that of the lowest mature process step.

It has to be put as a disclaimer that this work does not aim and thus cannot cover all topics relevant for the production of MEMS devices. Many more important aspects have been omitted, e.g., the reliability of the final devices [80,81], focusing only on the ones relevant for the proposed adoption to a research endeavor. From a technological perspective, the aspects of packaging and system integration have been neglected, which play a crucial role during MEMS development. They are best considered right from the beginning of the development journey, as they can lead to requirements and boundary conditions significantly influencing the device design and fabrication process [37,49]. The use of available established solutions—where possible—allows reducing complexity and helps focus development efforts on the relevant parts.

## 4. Conclusions

The demand for sensor fabrication within a collaborative research project led to exploring what can be learned from a production environment. We found that even in the dynamics of a research project, it is highly beneficial to adapt and apply tools and methods practiced in volume production. The key step is to change the perspective from fabricating devices to developing, maintaining and advancing the fabrication process. Using the teachings of process integration while carefully considering the intricate entanglement of device design and fabrication, the research and development of a MEMS device can both be sped up in time and reduced in efforts. Among the useful tools and methods discussed in this work, FMEA as a well-established risk assessment and mitigation concept can be a helpful frame guiding the development efforts. A systematic approach to change management plays an important role in handling the technological complexity.

However, the limitations of this approach have to be kept in mind as well, and care has to be taken adapting tools and methods to the specific situation. In particular, additional efforts have to be balanced against gained benefit, whose assessment is complicated by the fact that there is a significant delay between the investment and its payoff. The choices and decisions made in the example shown for illustration purposes are by no means binding and will probably differ depending on the particular situation.

In conclusion, the experiences made in this project confirm our view, and we will continue using, refining and amending the presented approach and workflow. While there may already be researchers and institutes using the proposed methods and tools in the best possible way, to the best of our knowledge, we sense a lot of room for improvement by systematically addressing these challenges. We strongly encourage looking beyond well-worn paths—not only from a technological but also from an organizational perspective—to further evolve MEMS research and development.

## Figures and Tables

**Table 1:** Preliminary specifications of the envisioned bulk micromachined delta-E effect ME sensor at the beginning of development.

Characteristic	Value
Physical size of sensor die	<10 mm × 10 mm
Limit of detection	<$100\;\mathrm{pT}/{\mathrm{Hz}}^{0.5}$ at 10 Hz
Sensitivity (shift of resonance frequency)	>10 Hz/mT at 10 Hz
Resonance frequency of first bending mode	1 kHz to 10 kHz
Electrical interfacing	pads to contact top and bottom electrodes of piezoelectric layer by wire bonding
Electrical capacitance	10 pF to 500 pF

**Table 2:** Excerpt of the D-FMEA for the developed ME sensor adapting the guidelines of [82].

#	Element/Function	Failure Effect (FE)	Failure Mode (FM)	Failure Cause (FC)	Prevention Measure (PM) for FC	Detection Measure (DM) for FC/FM	Severity (S) of FE	Occurrence (O) of FC	Detection (D) of FC/FM	Action Priority (AP)	Mitigation Measure (MM)
1	mechanical layer (poly-Si)	decreased sensitivity of sensor	cantilever too stiff	wrong thickness or material properties assumed	theoretical modeling, preexisting results	device characterization	7	5	3	med.	
2		incompatible with read-out circuitry	resonance frequency not in-spec	wrong thickness or length	theoretical modeling, preexisting results	device characterization	6	5	3	low	
3		device unusable	cantilever fractures	missing encapsulation during assembly	precaution	obvious	8	7	4	high	additional training
4	bond pads (metal contact layer)	device unusable	contact pads cannot be wire bonded	incomplete resist strip in fabrication		optical inspection in fabrication	8	4	6	med.	improve resist strip process

**Table 3:** Excerpt of the P-FMEA for ME sensor fabrication focusing on the contact metal layer adapting the guidelines of [82].

#	Process Step	Failure Effect (FE)	Failure Mode (FM)	Failure Cause (FC)	Prevention Measure (PM) for FC	Detection Measure (DM) for FC/FM	Severity (S) of FE	Occurrence (O) of FC	Detection (D) of FC/FM	Action Priority (AP)	Mitigation Measure (MM)
1	sputter deposition of AlCu	higher resistance or bad contact	deviation to lower thickness	design outside process window	process qualification	electrical test structure	8	4	7	high	evaluate and monitor test structures
2		bad contact to electrodes, electrical losses	high contact resistance or missing contact	oxide layer incompletely removed before deposition	optical inspection, in situ surface conditioning	test structure and 100% device measurement	7	5	4	med.	monitor test results
3	lithography of contact metal layer	no contact to electrodes, rework necessary	misalignment too large	inappropriate alignment marks		optical inspection	5	6	6	med.	
4	wet chemical structuring of AlCu	contact lines too thin, electrical losses, bad contact	underetching of AlCu	overetching, delamination of resist mask	lithography optimized for wet etching	test structure and 100% device measurement	8	5	8	high	design change contact line width or use dry etching process

## Data Availability

Not applicable.

## Abbreviations

The following abbreviations are used in this manuscript:

AP	action priority
CMOS	complementary metal-oxide semiconductor
CMP	chemical–mechanical polishing
CRC	collaborative research center
D	detection
DM	detection measure
DRC	design rule check
DRIE	deep reactive ion etching
FC	failure cause
FE	failure effect
FM	failure mode
FMEA	failure mode and effects analysis
GPL	GNU General Public License
IBE	ion beam etching
LPCVD	low-pressure chemical vapor deposition
ME	magnetoelectric
MEMS	microelectromechanical system
MES	manufacturing execution system
MM	mitigation measure
O	occurrence
PCM	process control monitor
PECVD	plasma-enhanced chemical vapor deposition
PM	prevention measure
RF	radio frequency
RIE	reactive ion etching
S	severity
TMAH	tetramethylammonium hydroxide

## Appendix A

In this section, the developed process flow for the fabrication of ME sensors is described in detail, relying on the cross-sections in Figure 2 for illustration.

The process flow starts with the fabrication of poly-Si substrates (Figure 2a). First, 1000 nm ${\mathrm{SiO}}_{2}$ is wet thermally grown on single-side polished 725 µm thick (100)-oriented Si wafers, which is followed by LPCVD deposition of polycrystalline Si that is polished down to 50 µm thickness using CMP. After polishing the wafer back side in another CMP step, 650 nm thick ${\mathrm{SiO}}_{2}$ is deposited on both sides in a high-temperature LPCVD process, which is followed by annealing at 1000 °C for 1 h. The piezoelectric layer stack is deposited on the substrates consisting of a 100 nm AlN seed layer, 100 nm Mo bottom electrode, 2000 nm AlN functional layer and 100 nm Mo top electrode using RF magnetron sputtering (Figure 2b). The Mo top electrode is wet chemically etched in phosphoric acid to act as a hard mask for structuring the AlN functional layer in TMAH (Figure 2c). Top and bottom Mo electrodes are structured to their final geometry in the next step (Figure 2d), repeating the wet chemical etching in phosphoric acid. A 1000 nm thick ${\mathrm{SiO}}_{2}$ isolation layer is deposited using PECVD and structured using an RIE process (Figure 2e). The structured isolation layer is used as a hard mask to remove the remaining AlN seed layer in TMAH. Having passed this step, the hard mask on the wafer back side for the later release etch is prepared by depositing an additional 500 nm SiN layer by means of PECVD and using RIE processes to open SiN and ${\mathrm{SiO}}_{2}$ layers on the back side (Figure 2f). A 800 nm thick AlCu layer is sputter deposited on the wafer front side to contact the electrodes through vias etched in the isolation layer and structured wet chemically using phosphoric acid (Figure 2g). The back side release is performed by KOH and TMAH etching in a single-side etching holder to protect the wafer front side (Figure 2h). The etching stops on the buried ${\mathrm{SiO}}_{2}$. Subsequently, the 200 mm wafers are diced to 50 mm square substrates. The magnetostrictive layer system is sputter deposited into the back side cavities and structured by IBE, which is followed by the sputter deposition of a 40 nm thick Ta layer to protect against corrosion (Figure 2i). The final fabrication step is etching the ${\mathrm{SiO}}_{2}$ and poly-Si layers using RIE and DRIE processes from the front side (Figure 2j) before singulation to 7 mm by 7 mm dies using saw dicing.

## Appendix B

In the following, the exemplarily shown test structures in Figure 3a are described in detail, ordered from left to right and top to bottom.

- AlN plate capacitor with contacts to top and bottom electrode to measure capacitance and loss tangent, sensitive to AlN film quality and thickness.
- Isolation ${\mathrm{SiO}}_{2}$ plate capacitor with contacts to bottom electrode and metal layer used as second electrode to measure capacitance and leakage current, sensitive to ${\mathrm{SiO}}_{2}$ film quality and thickness.
- Daisy chain of 36 contact vias to the top electrode to measure four-point-probe resistivity, sensitive to incomplete via opening, particles and other defects.
- Single contact via to the top electrode to measure four-point-probe resistivity, sensitive to contact resistance of top electrode and metal layer.
- Sheet resistance structure in metal layer to be measured by van-der-Pauw method, sensitive to metal layer quality and thickness.
- Finger structure in metal layer to measure leakage current, sensitive to incomplete structuring and other defects in metal layer.
- Meander structure in metal layer to measure four-point-probe resistivity, sensitive to metal layer quality, structuring resolution and defects.
- Meander structure in metal layer on top of topography to measure four-point-probe resistivity, sensitive to influence by topography (shorts and disconnects).

## Appendix C

Table A1, Table A2, Table A3 contain the evaluation criteria for the D- and P-FMEA presented in Table 2 and Table 3, as adopted from the guidelines published by the automotive industry [82].

**Table A1:** Severity criteria used for D- and P-FMEA, adopted from [82].

S	D-FMEA	P-FMEA
10	impact on safety and/or health of persons	
9	non-compliance with legal or regulatory requirements	
8	loss of a main function	discard whole batch
7	limitation of a main function	discard parts of the batch or lower yield
6	loss of a convenience function	whole batch undergoes additional rework steps
5	limitation of a convenience function	parts of the batch undergo additional rework steps
4	obvious impairment of quality	whole batch is reworked in the same step
3	impairment of quality	parts of the batch are reworked in the same step
2	hardly perceptible quality impairment	minor difficulty for process/tool/operator
1	no perceptible effect	

**Table A2:** Occurrence criteria used for D- and P-FMEA, adopted from [82].

O	Probability	D-FMEA	P-FMEA
10	≥10%	first-time application of new technology	no prevention measures
9	5%	first-time application of design with technical innovations; new material in company	low effectiveness of PM
8	2%	first-time application of a design with technical innovations; new material in application	
7	1%	new design based on similar technology or materials	moderate effectiveness of PM
6	0.2%	similar to previous design using existing technologies and materials	
5	0.05%	minor change from previous design using proven technologies and materials	PM are effective
4	0.01%	almost identical design with short field experience	
3	0.001%	minor change to known design or new design with successfully completed test procedure	PM are highly effective
2	0.0001%	almost identical mature design with long experience of use	
1	-	failure cause eliminated by design	PM are extremely effective, failure cause can physically not occur

**Table A3:** Detection criteria used for D- and P-FMEA, adopted from [82].

D	D-FMEA	P-FMEA
10	test method has not yet been developed	FM is not or cannot be detected
9	test method not specifically designed to detect FM or FC	FM cannot be detected in sampling test
8	new test method, not proven	manual inspection should detect FM or FC
7	new test method, not proven, but planning time sufficient to adjust design and/ or process	automatic measurement tools should detect FM or FC
6	proven test method, but planned use later in development cycle, negative results can lead to delays due to revisions	FM or FC is detected by manual inspection
5		FM or FC is detected by automatic measurement tools
4	proven test method; planned use is sufficient for adjustments and revisions	automatic measurement in a subsequent step marks product as defective
3		automatic measurement in this step marks product as defective
2		automatic detection of FC and prevention of FM occurrence
1	prior testing confirms that FM or FC cannot occur or is always detected by DM	FM physically cannot be caused or DM always detect FM or FC
